# 妊娠期急性白血病的诊治

**DOI:** 10.3760/cma.j.issn.0253-2727.2022.01.020

**Published:** 2022-01

**Authors:** 楠 彭, 梅英 梁, 倩 江

**Affiliations:** 1 北京大学人民医院，北京大学血液病研究所，国家血液系统疾病临床医学研究中心，北京 100044 Peking University People's Hospital, Peking University Institute of Hematology, National Clinical Research Center for Hematologic Disease, Beijing 100044, China; 2 北京大学人民医院妇产科，北京 100044 Department of Obstetrics and Gynecology, Peking University People's Hospital, Beijing 100044, China

急性白血病（AL）在妊娠人群中发病率为1～2/10万，其中，急性髓系白血病（AML）约占2/3，急性淋巴细胞白血病（ALL）约占1/3[1]–[2]。妊娠期出现AL是产科急危重症，AL诊断后若不治疗，孕妇可能在1～2个月内死亡[3]，诊断后应立即开始救治。国内外多项研究证实，妊娠期AL患者治疗后完全缓解（CR）率和预后与非妊娠患者相似[4]–[7]。妊娠合并AL的临床处理充满挑战，需要多学科共同协作，在维护产妇和胎儿健康的同时，对AL及并发症提出最佳决策，本文对相关文献和专家意见进行综述。

一、诊断

妊娠期常见症状（如乏力和气促）或血常规改变（如WBC升高、贫血和血小板减少）与AL症状重叠，可能导致AL诊断和治疗延迟[1]。外周血涂片中原始细胞升高提示白血病的可能，需完善骨髓穿刺检查。妊娠期可以进行骨髓穿刺和骨髓活检[8]，并常规完善血常规、外周血涂片、维生素B_12_、叶酸、铁蛋白、凝血功能、肾功能和肝功能检查。

妊娠患者AL诊断标准与非妊娠患者相同，依据WHO造血和淋巴组织肿瘤分类[9]。当疑诊白血病时，须完善骨髓细胞形态学、免疫分型、细胞遗传学和分子生物学检测，以明确诊断和分型。

二、治疗

妊娠期AL治疗方案的制定受胎龄、AL类型、孕妇病情、胎儿情况、患者及家属对母胎的态度等多种因素影响。AL增加孕妇弥散性血管内凝血（DIC）（7.6％）、产后出血（7.2％）、静脉血栓栓塞（5.8％）、孕产妇死亡（4.1％）等风险，同时增加孕妇血制品输注（33.3％），以及早产（14.78％）、宫内胎儿死亡（2.06％）等胎儿并发症[2]。因此，患者治疗前需多学科协作评估治疗对孕妇和胎儿的风险及获益，并与患者和家属充分沟通，共同决策治疗方案。

1. 治疗原则：妊娠期AL患者的治疗策略通常取决于诊断AL时的孕期：妊娠早期（≤12周）、妊娠中期（13～27^+6^周）、妊娠晚期（≥28周）。

在妊娠早期，化疗可致自然流产、死胎和严重胎儿畸形的风险增加10％～20％[10]–[11]。此期接受化疗，最重要的危害是化疗药物可能导致胎儿畸形[3],[12]–[13]，还可抑制妊娠早期人胎盘滋养细胞迁移和增殖，可能导致低出生体重儿[14]。根据国际共识会议的管理指南、英国血液学标准委员会、德国癌症研究中心、Brenner、Milojkovic等推荐[12],[15]–[16]，妊娠早期必须与患者及家属充分沟通治疗性流产的必要性，应首先建议治疗性流产终止妊娠。当孕妇血小板和凝血功能在安全范围内，宜择期终止妊娠[17]。通常推荐血流动力学稳定的患者在妊娠早期终止妊娠后，宜尽快接受治疗，方案同非妊娠患者[10],[15]。若病情危重且发生DIC，应在支持治疗的同时开始诱导治疗，待病情稳定后再终止妊娠[18]–[19]。

在妊娠中晚期，化疗可能相对安全，化疗致胎儿先天畸形风险仅约3％。尽管会增加胎儿宫内发育迟缓、死胎等风险[3],[16],[20]，但化疗导致妊娠中期（9.7％）及晚期（0％）死胎和自然流产发生率低于妊娠早期（37.5％）[21]。根据英国血液学标准委员会推荐，在患者及家属、产科医师、血液科医师和新生儿医师仔细讨论化疗和分娩时机后：①妊娠13～24周诊断的AML患者，应考虑开始诱导治疗并允许继续妊娠。②妊娠24～32周诊断的AML患者，需评估胎儿暴露于诱导治疗的风险与择期分娩早产风险的利弊[12]。天津医科大学总医院张慧英等[18]建议，如已发生DIC，除非出现产科急症或近预产期，尽可能先诱导治疗，纠正DIC后再终止妊娠。③妊娠期超过32周后诊断的AL患者，若一般情况尚好，并无DIC表现，一旦胎儿成熟，产科医师应尽快制定择期分娩计划，成功分娩后再开始诱导治疗。④妊娠36周后应避免化疗，建议择期分娩[12],[16]–[18]。剖宫产或阴道分娩后尽快予后续治疗，治疗方案同非妊娠患者。

2. AL常用药物对母胎影响：任何细胞毒性药物可致母亲和胎儿可逆性全血细胞减少和脱发，避免用于妊娠35周后或计划分娩2～3周内[13]；宫内暴露于细胞毒性药物后，早产（11.8％）和小于胎龄儿（24.2％）比例增加[22]。治疗AL的药物对母胎的影响和建议见表1。

**表1:** 治疗急性白血病的药物对母胎的影响和专家建议[3],[12]–[13],[15]–[16],[23]–[30]

药物	疾病	影响	建议
阿糖胞苷	AML、ALL	妊娠早期：先天畸形（常见肢体和耳畸形） 短暂血细胞减少、宫内发育迟缓、宫内死胎和脓毒症致新生儿死亡，风险较小	妊娠中晚期：治疗同非妊娠患者
蒽环类药物	AML、ALL	妊娠早期：肢体畸形 妊娠中晚期：子痫前期，宫内发育迟缓，宫内死胎，流产（尤其伊达比星） 伊达比星可致新生儿心肌病	阿霉素：ALL首选 柔红霉素/表柔比星：AML推荐；ALL次选 伊达比星：禁忌 产前胎儿心脏超声监测心脏毒性
羟基脲	AML	动物研究：先天畸形 人体研究：自然流产	不建议用 初诊WBC≥100×10^9^/L，化疗前用可能改善预后
砷衍生物	APL	动物研究：严重胚胎毒性和致畸性	禁用
全反式维甲酸	APL	致畸率85％，胎儿严重颅面、心脏、中枢神经系统和胸腺异常及流产率增加	妊娠中晚期：治疗同非妊娠患者
门冬酰胺酶	ALL	动物研究：胎儿骨骼畸形 人体研究：暂无单药数据，联合化疗可致低出生体重儿；可能增加血栓风险	仅在有明确指征且获益大于风险时谨慎使用
糖皮质激素（地塞米松、泼尼松）	ALL	动物研究：妊娠早期，口唇裂风险增加3.4倍 人体研究：胎膜早破、妊娠糖尿病和高血压风险增加	监测新生儿是否出现肾上腺功能不全和感染
环磷酰胺	ALL	单药使用，非特异性致畸率55％	妊娠中晚期：治疗同非妊娠患者
甲氨蝶呤	ALL	妊娠早期：先天畸形和流产风险增加 妊娠中晚期：剂量>10 mg/周时，氨蝶呤综合征（颅骨发育不全、眶距增宽、鼻梁宽、骨化延迟、小颌畸形和耳畸形）	胎龄20~28周：不用 妊娠晚期：治疗同非妊娠患者
6-巯嘌呤	ALL	单药治疗风险未知 妊娠早期联合化疗致畸率30％	妊娠早期：谨慎使用 妊娠中晚期：治疗同非妊娠患者
酪氨酸激酶抑制剂	ALL	动物研究：伊马替尼、尼洛替尼和达沙替尼具有胚胎毒性；伊马替尼和达沙替尼可致先天畸形 人体研究：流产和先天畸形（肾脏、消化道、脑、心脏及骨骼），妊娠早期风险最高	妊娠早期：禁忌； 妊娠中晚期：尽可能避免
长春新碱	ALL	无先天畸形或神经毒性报道	证据缺如

注：AML：急性髓系白血病；ALL：急性淋巴细胞白血病；APL：急性早幼粒细胞白血病

3. 妊娠期各种类型AL的治疗：

（1）AML（非APL）：妊娠早期诊断的AML患者终止妊娠后，诱导化疗方案同非妊娠患者[10]。

妊娠中晚期诊断的AML患者推荐柔红霉素（60 mg/m^2^第1、3、5天）联合阿糖胞苷（100 mg/m^2^持续输注第1～7天或第1～10天）作为标准诱导化疗方案，根据孕妇实际体重进行剂量调整，定期监测胎儿心脏功能和是否发生先天性发育异常[1],[12],[16]。Horowitz等[5]对138例妊娠期AML患者进行系统评价，对于接受阿糖胞苷联合蒽环类药物为基础方案的患者，妊娠中期、晚期和产后接受治疗的CR率分别为83％（29/35）、80％（8/10）和100％（12/12），高于其他诱导方案；接受该方案患者OS率为79％，不低于非妊娠者；活产率为86.8％（46/53）；宫内暴露于化疗的新生儿中，低出生体重儿为36％，并发症发生率为21％（12/58），包括畸形4例（动脉导管未闭、脑积水、室间隔缺损各1例，未指明1例），血液学并发症3例（贫血、中性粒细胞减少、母体血型不合免疫溶血性贫血各1例），神经系统不良事件2例（颅内出血、抽搐各1例），呼吸系统并发症5例（肺炎或呼吸窘迫），脓毒症和肝肿大1例，颅内出血导致胎儿死亡1例。Chang等[21]系统评价83例妊娠期AML患者及其85例胎儿，宫内胎儿均暴露于阿糖胞苷，47例暴露于柔红霉素，8例暴露于伊达比星，阿糖胞苷联合柔红霉素引发胎儿缺陷率和死胎率分别为8.5％（唐氏综合征、9号染色体倒位、先天性左眼虹膜与后角膜粘连、尿道下裂各1例）和6.4％，而阿糖胞苷联合伊达比星分别高达28.6％（肢体缺陷2例）和12.5％，故推荐阿糖胞苷联合柔红霉素用于妊娠中晚期诊断的AML患者。

关于移植，中危和高危AML患者，若有合适供者，应按分娩后尽早行异基因造血干细胞移植的方案安排治疗和分娩[5]。

（2）APL：全反式维甲酸（ATRA）和三氧化二砷（ATO）均有强致畸性。在妊娠期发生APL相当罕见，仅有少量回顾性研究和病例报道。

若患者高度怀疑APL或出现DIC或高白细胞血症，不论胎龄，尽早予以ATRA，病情稳定后治疗性流产[31]–[32]。

在妊娠早期，APL患者终止妊娠后，诱导治疗方案同非妊娠患者[15]。

在妊娠中晚期，低中危APL患者推荐ATRA单药诱导治疗，高危APL或无法通过实时定量PCR监测PML-RARα水平的患者推荐ATRA联合柔红霉素为基础的化疗[15]。在系统评价71例妊娠期APL患者中，诱导治疗包括ATRA、蒽环类药物和阿糖胞苷的各种方案，58例可评估患者中93.1％达CR；母胎并发症包括早产（46％）、自然流产/治疗性流产/宫内死胎（33.3％）和其他新生儿并发症（25.9％）；ATRA组与非ATRA组的胎儿并发症发生率相当［43.3％（13/30）对20.0％（2/10），*P*＝0.27］[31]。汇总三项国内研究，11例妊娠中晚期低中危APL患者在终止妊娠前采用ATRA单药治疗，9例CR，2例部分缓解，10例胎儿均存活，无先天畸形，1例治疗性流产、无先天畸形[32]–[34]。分娩后宜尽快后续治疗，高危APL患者接受ATRA联合柔红霉素及阿糖胞苷化疗，非高危APL患者接受ATO联合ATRA[15]。

妊娠中晚期接受ATRA治疗的孕妇，宜密切监测胎儿心功能指标，因有报道可逆性胎儿心律失常和其他心脏并发症[27],[35]–[36]。妊娠期APL患者的早产儿中，最常见新生儿并发症为呼吸窘迫综合征（12/47），除2例分娩后早期死亡（Potter's综合征和肺出血），其余新生儿均预后尚可[6]。

（3）ALL：妊娠期ALL治疗的报道非常有限。根据国际共识会议的管理指南、Brenner、Milojkovic等推荐[10],[14],[16]：①妊娠20周前，应终止妊娠，之后开始常规化疗。因为妊娠早期ALL患者化疗可导致自然流产、肢体畸形、肺部畸形、食道闭锁、死胎及继发肿瘤等严重不良结局[37]。②妊娠邻近20周，建议单用泼尼松1～2周，使患者进入妊娠20周后接受化疗。③妊娠20周后，采用化疗方案同于非妊娠ALL患者，但不宜使用甲氨蝶呤，直到妊娠晚期。④妊娠邻近32周，建议单独采用泼尼松的类似方案，直到分娩。

对于妊娠中晚期诊断ALL的患者接受化疗的结局，Ticku等[38]对17例患者进行文献综述，大部分方案包含蒽环类药物、长春新碱和糖皮质激素，12例缓解，3例复发，5例死于病情恶化；胎儿和新生儿并发症包括死胎、宫内发育迟缓、高胆红素血症、呼吸窘迫和早产各1例，短暂全血细胞减少2例。Zaidi等[13]的研究显示，妊娠中晚期ALL患者接受标准联合化疗对胎儿长期生长发育无严重影响，但妊娠中晚期胎儿暴露于化疗药物常与宫内发育迟缓和低出生体重儿相关。

Ph^+^ALL少有妊娠期报道。Mainor等[39]报道1例妊娠24周的Ph^+^ALL患者，采用hyperCVAD（环磷酰胺、地塞米松、阿霉素、长春新碱）联合伊马替尼治疗，孕30周剖宫产一低出生体重儿，Apgar评分5和9，无先天异常。患者产后第3疗程化疗期间出现小肠梗阻、心跳骤停，予恢复自主循环、广谱抗生素、肾替代治疗等，产后7周死于脆弱类杆菌败血症。由于目前证据有限，建议Ph^+^ ALL患者产后使用酪氨酸激酶抑制剂[10]。

三、分娩的准备和管理

对AL孕妇的管理需要包括血液科、产科、新生儿科和麻醉医师以及护士的多学科团队良好协作[2]。

选择分娩时间应避免在孕妇血细胞计数最低点（通常为化疗后2～3周），以减少出血和感染风险，也降低新生儿骨髓抑制风险，尤其早产儿可通过胎盘排出药物避免肾脏负担[13]。

阴道分娩优于剖宫产，因为前者感染和出血风险更低、身体恢复更快，且不延误后续治疗[16]。通常，PLT≥50×10^9^/L且凝血功能正常可行阴道分娩。如有产科指征，应择期进行剖宫产，尤其是初产妇，以缩短产程、减少产妇消耗及第二产程屏气用力、避免脑出血及软产道损伤，但需PLT≥80×10^9^/L且凝血功能正常。

麻醉团队尽早参与镇痛方案，若孕妇WBC<1×10^9^/L或PLT<80×10^9^/L，不推荐硬膜外镇痛，以减少血肿和感染的风险，可应用哌替啶。若PLT<50×10^9^/L，应避免肌肉注射。若中性粒细胞减少或血小板减少的孕妇需要剖宫产，应在全身麻醉下进行[12]。

根据英国血液学标准委员会、欧洲白血病网专家组和英国皇家妇产科学院的建议[12],[27]，糖皮质激素（如地塞米松、倍他米松）宜在妊娠24～35周早产前使用，以减少早产相关风险（包括呼吸窘迫综合征、脑室出血、坏死性小肠结肠炎和脑瘫等）。糖皮质激素应在计划性早产前一周内给予48 h。

分娩后，在母亲接受化疗期间以及化疗后的2周内，应避免母乳喂养；对于接受巩固和维持治疗的AL患者，应采取有效的避孕措施[12],[15],[27]。

四、支持治疗

1. 血制品：妊娠期患者输血指征同于非妊娠患者[40]。

2. 止吐药：根据英国畸形信息服务（UK Teratology Information Service, UKTIS），首选止吐药物包括抗组胺药、苯甲嗪和异丙嗪；丙氯拉嗪和甲氧氯普胺是二线药物，因为可能与孕妇的肌张力障碍相关（UKTIS 2010）。

3. 抗感染药物：青霉素类、氨基糖苷类、甲硝唑和头孢菌素类药物在妊娠期可安全使用。喹诺酮类、四环素类、磺胺类药物和阿莫西林克拉维酸钾应避免使用[41]。两性霉素B是在妊娠期应用最广泛的抗真菌药物，目前尚无致畸报道[16]。产褥期应采用抗生素预防感染。

4. G-CSF：G-CSF能在妊娠中晚期透过胎盘[42]。基于有限数据，妊娠期使用G-CSF似乎对母胎无明显危害[3],[14]。

5. 妊娠期APL患者临床可疑早期诱导分化综合征时，治疗方案基本同非妊娠APL患者[40]。

五、新生儿随访

儿科团队的保障及随访对于减少婴儿任何潜在不良影响是必不可少的。新生儿骨髓抑制风险总体较低，通气管和产钳不是禁忌[12]。胎盘娩出前注意留取脐带血，做白血病相关筛查[19]。McReynolds等[43]报道杂合致病种系CEBPA变异是遗传性AML易感病因，约100％外显率；杂合致病种系PAX5变异可致家族性ALL；一级亲属患AL（尤其是年龄小于45岁）的儿童或年轻AL患者，应检测相关基因。

妊娠期母亲接受化疗对子代是否存在长期不良影响需随访，特别是神经发育、生育能力及继发性恶性肿瘤情况[22]。Avilés等[44]对84例宫内暴露于化疗的儿童进行长达19年的随访，儿童神经发育、学习成绩、性成熟和生殖能力均正常。其中，母亲曾患AL的29例儿童（中位年龄11.4岁）均未发生恶性肿瘤。

六、具有AL病史患者的后续妊娠结局

AL患者经过治疗获得康复后，若在育龄期希望生育，需关注妊娠和子代的结局。Anderson等[45]发现，与普通人群相比，诊断时小于40岁的患者治疗后妊娠可能性降低52％，在30年随访期间，白血病患者治疗后妊娠率持续很低，骨髓移植前接受全身照射或高剂量烷化剂可增加丧失生育能力的风险。在系统评价研究中，与非癌症患者相比，具有AL病史的患者妊娠后自然流产（6％～14％）、死产（1.2％）、胎儿出生缺陷（3.6％）、子代患癌症（1.7％）的风险未增加，子代性别比例（男∶女，0.9～1.4）无明显变化，但活产率（62％）低于健康对照（71％），早产（19％对7％～13％）和低出生体重儿（9％对4％～8％）的风险高于健康对照[46]。Brenner等[14]建议，持续缓解不足2～3年的AL患者应避免妊娠，因为此时AL复发的可能性较高，但妊娠是否增加复发风险尚无证据。

综上所述，妊娠期发生AL在临床罕见，母胎存在极大风险，治疗需要多学科参与，与患者及家属充分沟通，结合胎龄、孕妇病情、胎儿情况和AL类型等因素，共同制定个性化治疗方案，从而减少母儿并发症，提高活产率，改善妊娠结局。
